# Brain-computer interfaces and neural synchronization in esports: a systematic review of effects on reaction time, decision-making, and cognitive performance

**DOI:** 10.3389/fnhum.2026.1774230

**Published:** 2026-05-08

**Authors:** Prashant Kumar Choudhary, Suchishrava Choudhary, Sohom Saha, Yajuvendra Singh Rajpoot, Vasile-Cătălin Ciocan, Voinea Nicolae-Lucian, Carmina Mihaela Gorgan, Constantin Șufaru

**Affiliations:** 1 Department of Physical Education Pedagogy, Lakshmibai National Institute of Physical Education, Gwalior, India; 2 Department of Sport Psychology, Lakshmibai National Institute of Physical Education, Gwalior, India; 3 Department of Sports Management and Coaching, Lakshmibai National Institute of Physical Education, Gwalior, India; 4 Faculty of Movement, Sports, and Health Sciences, “Vasile Alecsandri” University of Bacău, Bacău, Romania

**Keywords:** brain-computer interfaces, decision-making, esports performance, motor imagery, neural synchronization, reaction time, visual evoked potentials

## Abstract

**Background:**

The rapid expansion of esports has intensified interest in the cognitive and neurophysiological mechanisms underlying elite performance, particularly reaction time (RT), decision-making (DM), and neural efficiency. Advances in brain-computer interfaces (BCIs) offer targeted neural modulation that may enhance these abilities through improved neural synchronization. To systematically review evidence on the effects of BCI-based neural synchronization, including motor imagery (MI) BCIs, visual evoked potential (VEP/c-VEP) systems, neural entrainment, and dual-brain coupling, on RT, DM, and related cognitive outcomes in esports athletes and competitive gamers.

**Methods:**

Following PRISMA 2020 guidelines, comprehensive searches were conducted across PubMed, Scopus, Web of Science, IEEE Xplore, PsycINFO, ScienceDirect, and Google Scholar. Studies examining BCI-induced neural modulation and its cognitive or performance effects in esports players or experienced gamers were included. Eighteen studies met the criteria, comprising controlled trials, pre–post interventions, cross-sectional neurophysiology studies, comparative behavioural analyses, and supporting systematic reviews. Due to methodological heterogeneity, results were synthesised narratively. Although the review follows PRISMA 2020 guidelines for systematic study identification and selection, the synthesis adopts a structured integrative narrative approach due to substantial heterogeneity in study designs, BCI modalities, and outcome measures.

**Results:**

Across studies, BCI-mediated neural synchronization produced consistent improvements in RT, DM accuracy, cortical oscillatory stability, and neural connectivity. MI-BCI and gamified systems enhanced MI accuracy, user engagement, and cognitive load regulation. VEP-based BCIs accelerated perceptual processing by improving signal reliability and reducing latency. Dual-brain coupling improved coordinated decision behaviour. Additional evidence indicates that experienced gamers display superior working memory, attentional control, and visuomotor coordination compared with non-gamers. However, variability in study design, small samples, and moderate risk of bias limit the strength of causal inference.

**Discussion:**

BCI-based neural synchronization shows promise as a tool for enhancing neurocognitive performance in esports athletes. Future studies should prioritize standardized training protocols, multimodal neural-measurement methods, and longitudinal designs to determine long-term effectiveness and real-world applicability.

## 1 Introduction

In recent years, video gaming has evolved into one of the most widespread forms of entertainment, extending far beyond its traditional association with children and adolescents. Demographic trends indicate that the average age of video game players has steadily increased, reaching 33 years by 2008 (Williams et al., 2008), reflecting the growing involvement of adults in gaming activities. The rapid expansion of digital technology, particularly smartphones and tablets, has further contributed to this shift, exposing large segments of the population to casual games and gamified applications. Mobile games are characterized by their portability, emphasis on short events, easy-to-learn gameplay, and freemium models, making them highly accessible (Bowman et al., 2012). Despite the popularity of gaming, public discourse continues to present conflicting claims regarding its health implications, often driven by sensationalized media coverage lacking a scientific foundation. With continuous advancements in hardware and software, video games have become more sophisticated and appealing to older audiences, leading to a significant rise in the number of individuals who spend substantial time engaging in digital gameplay daily. By analyzing cerebral activity without requiring muscular movement, BCIs enable direct communication between the human brain and external devices (Chaudhary et al., 2016; Saha et al., 2021; Kawala-Sterniuk et al., 2021; Wolpaw and Wolpaw, 2012; Abibullaev et al., 2023). Initially developed to support individuals with severe motor impairments resulting from conditions such as amyotrophic lateral sclerosis (ALS) or spinal cord injuries, BCIs have advanced considerably over the past several decades (Chaudhary et al., 2016; Kawala-Sterniuk et al., 2021; Wolpaw and Wolpaw, 2012). Progress in neuroscience, signal processing, and machine learning has broadened their applications beyond medical rehabilitation into domains such as education, entertainment, human-computer interaction, and telepresence robotics (Chaudhary et al., 2016; Saha et al., 2021; Kawala-Sterniuk et al., 2021; Keutayeva and Abibullaev, 2024; Saduanov et al., 2018).

Within competitive esports an arena characterized by extreme cognitive demands BCIs have emerged as a promising tool for enhancing neural efficiency, reaction time, and decision-making under pressure. Several studies highlight that targeted neural modulation can synchronize cortical oscillations and improve performance-relevant cognitive functions. For instance, transcranial neural entrainment techniques, including transcranial alternating current stimulation (tACS) and other non-invasive brain stimulation (NIBS) methods, have been shown to enhance reaction time and decision accuracy (Colzato et al., 2017). Within the gaming context, parietal alpha and beta oscillations have been linked to superior competitive performance among esports athletes, suggesting that neural synchronization plays a critical role in high-level gaming outcomes (Minami et al., 2023). Other work demonstrates that winners of BCI-based competitions exhibit stronger directed fronto-parietal connectivity, highlighting the relationship between neural coherence and decision-making efficacy (Putri et al., 2022). In the present review, the term “neural synchronization” is used in a broad but structured sense to encompass multiple related neurophysiological processes, including oscillatory coherence, phase synchronization, functional connectivity, and, in some contexts, inter-brain coupling. While these phenomena are conceptually linked through their association with coordinated neural activity, they represent distinct levels of analysis and measurement. Accordingly, the review adopts a cautious interpretative framework, recognizing that these mechanisms may not reflect a single unified process but rather a family of related neural coordination dynamics.

A growing body of research also emphasizes the impact of Motor Imagery-BCI (MI-BCI) systems, particularly when enhanced through gamification. Gamified MI-BCI environments have been shown to accelerate learning, reduce cognitive load, and improve neural engagement (Atilla et al., 2024; Gao et al., 2022). Experimental MI-BCI training has also produced significant improvements in reaction time and motor control accuracy (Yakovlev et al., 2020). Beyond MI-BCI, dual-brain coupling BCIs have demonstrated that synchronized neural states between users improve cooperative performance accuracy, suggesting new possibilities for team-based esports training (Jia et al., 2024). Complementary evidence from VEP-based BCIs indicates that enhanced signal quality and reduced latency further contribute to faster perceptual processing (Keutayeva et al., 2025).

Cognitive enhancements associated with gaming and esports training have also been documented outside of direct BCI use. Gamers consistently outperform non-gamers in reaction time, working memory, and attentional control tasks (Ziv et al., 2022). Large-scale observational research further highlights that esports participants exhibit strong baseline reaction times and sensorimotor coordination compared to the general population (Cyma-Wejchenig et al., 2024). Related studies exploring bias reduction and cognitive optimization indicate that BCI-based cognitive training can improve executive control and decision-making under uncertainty (Tmienova and Mykhalchuk, 2023). Additionally, VR-based cognitive-motor training, eye-tracking studies of expert fixations, and neural hemodynamic responses to high-intensity activity contribute further evidence that perceptual-cognitive performance can be modulated through targeted neural or sensorimotor training (Mancı et al., 2024b; Velichkovsky et al., 2019; Lachowicz et al., 2024).

Collectively, these studies underscore the potential of BCI technologies and neural synchronization training as emerging tools for enhancing reaction time, decision-making, neural efficiency, and cognitive readiness in esports. However, the rapid expansion of this field warrants a systematic evaluation of existing evidence to understand the extent to which BCIs contribute to performance enhancement and how neural synchronization processes relate to competitive gaming outcomes. Therefore, the present review aims to systematically identify and evaluate the available evidence on BCI-driven neural synchronization and its impact on reaction time, decision-making, cognitive performance, and neurophysiological efficiency in esports athletes and related gaming populations. While the study selection and screening procedures follow PRISMA 2020 guidelines to ensure methodological transparency and reproducibility, the synthesis adopts a structured integrative narrative approach due to substantial heterogeneity in study designs, BCI modalities, neural metrics, and outcome measures. Accordingly, the review is positioned as a systematic review with integrative synthesis, emphasizing conceptual interpretation and cross-study comparison rather than purely quantitative aggregation. While study identification and selection followed systematic procedures, the synthesis emphasizes conceptual integration of representative findings rather than exhaustive quantitative comparison across all included studies. This approach ensures that all interpretive conclusions remain grounded in systematically identified evidence, while allowing flexibility for conceptual integration across heterogeneous studies.

## 2 Materials and methods

### 2.1 Study design

This systematic review was conducted in accordance with the Preferred Reporting Items for Systematic Reviews and Meta-Analyses (PRISMA 2020) guidelines (Moher et al., 2009; Page et al., 2021). The review was designed to synthesize quantitative and experimental evidence from studies that investigated how neural synchronization training and BCI technologies influence reaction time, decision-making performance, and neurocognitive functioning in esports athletes and competitive gamers. The review employed a mixed-method evidence synthesis approach to accommodate the wide range of study designs found in BCI and esports neuroscience research, including randomized controlled trials, quasi-experimental interventions, pre-post training studies, controlled laboratory experiments, and observational cross-sectional investigations. Given the interdisciplinary nature of the topic, integrating neurophysiology, cognitive science, human-computer interaction, and esports performance, the review was structured to capture both neural outcomes (e.g., connectivity, oscillatory power, VEP responses, MI-BCI learning) and behavioural outcomes (e.g., reaction time, decision accuracy, task performance). The design incorporated a broad search strategy across multiple scientific databases, followed by systematic screening, eligibility assessment, data extraction, and methodological quality evaluation. No restrictions were placed on publication year to ensure that both foundational and emerging BCI methodologies were captured. The chosen design allowed for comprehensive comparison across heterogeneous methodologies while maintaining a consistent analytical framework for evaluating the impact of BCI-mediated neural synchronization processes within esports performance contexts. Importantly, although the review adheres to systematic principles of study identification, screening, and risk-of-bias evaluation, the synthesis phase is intentionally integrative and narrative in nature. This approach was necessary due to the heterogeneity of included studies in terms of experimental paradigms, neural synchronization metrics, and performance outcomes. Therefore, the review combines systematic methodology with interpretative synthesis, allowing both structured evidence appraisal and conceptual integration across diverse study designs.

### 2.2 Search strategy

This systematic review followed the PRISMA 2020 guidelines and employed a comprehensive electronic search across PubMed, Scopus, Web of Science, IEEE Xplore, PsycINFO, ScienceDirect, and Google Scholar, without restrictions on publication year, to identify studies examining neural synchronization training and BCI-based enhancement of reaction time and decision-making performance in esports or competitive gaming contexts. Search strings combined controlled vocabulary and free-text terms such as “brain-computer interface,” “BCI,” “neural synchronization,” “neurofeedback,” “motor imagery,” “VEP,” “c-VEP,” “EEG connectivity,” “reaction time,” “decision-making,” and “esports athletes,” using Boolean operators to maximize sensitivity. Reference lists of eligible studies and relevant systematic reviews were manually screened to capture additional sources. In addition to database searching, study selection was guided by conceptual relevance to the research objectives. Priority was given to studies that directly examined brain–computer interfaces, neural synchronization processes, or neurocognitive outcomes such as reaction time, decision-making, and cognitive control in esports or gaming contexts. This approach ensured that the review captured representative and theoretically relevant studies contributing to understanding the neural and cognitive mechanisms associated with esports performance.

### 2.3 Eligibility criteria

Studies were included if they met the following criteria: (a) involved human participants engaged in esports, competitive gaming, or BCI-dependent performance tasks; (b) employed a BCI modality such as EEG-based motor imagery, VEP or c-VEP, neural synchronization training, neurofeedback, or brain-to-brain coupling; (c) reported quantitative outcomes related to reaction time, decision-making accuracy, neural synchronization, cognitive performance, or BCI learning metrics; (d) were peer-reviewed full journal articles or full conference proceedings; and (e) were published in English. Studies were excluded if they focused solely on hardware development, lacked human cognitive or neural outcomes, were reviews, editorials, or commentaries, involved clinical populations unrelated to esports performance, or lacked full-text availability (Table 1).

**Table 1:** Inclusion and exclusion criteria for the systematic review.

Criteria category	Inclusion criteria	Exclusion criteria
Population	Participants involved in esports, competitive gaming, or BCI-based competitive tasks (FPS, MOBA, Strategy, Rhythm games, VR esports). Healthy adults or adolescents (≥12 years) relevant to esports cognitive profiles.	Clinical populations (e.g., neurological diseases, psychiatric disorders) unless related to BCI performance. Non-gaming populations with no competitive gaming or BCI-task relevance. Animal studies.
Intervention/Exposure	• Studies involving BCI systems, including: Motor Imagery EEG-BCI VEP/c-VEP BCI Neural synchronization training Brain-to-brain coupling systems Neurofeedback-based RT or DM training	Studies not involving any BCI system. Studies focusing solely on hardware development without human performance data. Studies involving brain stimulation without BCI integration unless linked to neural synchronization or RT/DM outcomes.
Primary Outcomes	Reaction time Decision-making accuracy (DM) • In-game decision or response performance	Studies not reporting any performance, RT, or decision-making outcomes. Studies focusing only on emotional, personality, or social outcomes without relevant cognitive measures.
Secondary Outcomes	Neural synchronization metrics: DTF, PLV, coherence, oscillatory amplitude Neural efficiency, cortical hemodynamics, visual fixation, cognitive load • MI-BCI learning accuracy or VEP latency	Studies reporting only subjective surveys or qualitative interviews with no neural/cognitive data.
Study Designs	Randomized controlled trials (RCTs) non-randomized controlled studies Pre-post intervention studies Observational studies (cross-sectional, comparative) Experimental BCI task-based studies Systematic reviews used for background justification (not included in analysis)	Editorials, opinion pieces, letters, commentaries Conference abstracts with insufficient data Protocol-only papers Reviews (excluded from quantitative synthesis but may support narrative background)
Language	Studies published in English	Studies published in languages other than English (without translation available)
Publication Type	Peer-reviewed journal articles Full conference papers with complete methodology	Theses, dissertations, posters, book chapters, unpublished manuscripts
Time Frame	No restriction on publication year (to capture foundational BCI/neural-synchronization work)	
Accessibility	Full-text available	Full-text not retrievable after reasonable attempts

### 2.4 Study selection

All records retrieved were imported into a reference manager, duplicates were removed, and two independent reviewers screened titles and abstracts for relevance. Full texts of potentially eligible articles were then assessed independently by both reviewers against the inclusion and exclusion criteria, with discrepancies resolved through discussion or a third reviewer. The screening process and final study numbers were documented using the PRISMA 2020 flow diagram.

### 2.5 Data extraction

A standardized extraction sheet was used to collect essential study information including author, year, country, study design, sample characteristics, esports/game genre, BCI system specifications, neural recording parameters (channels, sampling rate, connectivity metrics, oscillatory features), training protocol (session duration, frequency, feedback type), primary outcomes (RT, DM accuracy, BCI task accuracy), secondary outcomes (neural synchronization indices, cognitive performance, eye-hand coordination), and main findings. Two reviewers independently extracted data to ensure accuracy.

### 2.6 Risk of bias assessment

The methodological quality and risk of bias of the included studies were evaluated using a customized appraisal framework adapted specifically for the context of neural synchronization and BCI research in esports. Because the review included a mixture of experimental, quasi-experimental, and observational designs, a flexible yet rigorous approach was essential. We therefore drew upon the risk of bias structure used in prior interdisciplinary reviews of behavioural and cognitive science (Barker et al., 2024), which provides an appropriate foundation for assessing heterogeneous study types. However, given the unique methodological features of BCI-based research, such as neural signal validity, synchronization metrics, device calibration, and cognitive-behavioural outcome precision, we supplemented this framework with targeted criteria derived from mediation and mechanism-focused evaluations proposed by Pan et al. (2024), Cruz et al. (2025), and Gudikandula et al. (2025). These additions enabled a more comprehensive assessment of whether studies reported valid neural measures (e.g., EEG oscillations, connectivity indices, VEP latency), accurately captured behavioural outcomes (e.g., reaction time and decision-making accuracy), and applied appropriate statistical or signal-processing methods to support mechanistic interpretations. Each study was scored on domains covering participant eligibility, methodological transparency, neural measurement validity, BCI protocol quality, appropriateness of analytical procedures, and treatment of potential confounders such as gaming experience, fatigue, or learning effects. Items were scored dichotomously (1 = present, 0 = absent/inadequately described), and total scores were used to categorize studies as high quality (score ≥ 6–7), acceptable quality (score = 4–5), or low quality (score ≤ 3), consistent with the grading method outlined by De Cassai et al. (2025). Two reviewers independently applied the criteria and resolved disagreements through discussion until consensus was achieved, ensuring robust evaluation of risk of bias across all included studies.

### 2.7 Data synthesis

Due to heterogeneity in study designs, BCI systems, neural variables, and performance metrics, a narrative synthesis approach was used to summarize findings across domains, including reaction time, decision-making accuracy, neural synchronization, cognitive outcomes, and motor-cognitive integration. Studies were grouped according to outcome type and methodological similarity. Quantitative pooling (meta-analysis) was considered but not performed because outcome measures and neural synchronization metrics were not consistently comparable across studies. The synthesis, therefore, focuses on direction, magnitude, and consistency of effects across multiple methodological approaches (Table 2).

**Table 2:** Risk of bias criteria used for quality assessment.

No.	Quality assessment domains
1.	Participant eligibility is clearly defined, including gaming experience, esports level (amateur/elite), health status, and appropriateness of the sample for neural synchronization or BCI research.
2.	Power calculation or sample justification reported, including neural-signal reliability considerations and whether the study was adequately powered to detect changes in reaction time, decision-making, or neural synchronization metrics.
3.	Valid and reliable BCI or neural recording measures used, such as properly calibrated EEG systems (e.g., motor imagery, VEP/c-VEP, connectivity metrics), reporting of channels used, sampling rate, artifact processing, and signal quality indices.
4.	Valid performance outcome measures, including reaction time tests, decision-making tasks, in-game performance metrics, or cognitive assessments with acceptable reliability (e.g., test–retest reliability, internal consistency).
5.	Valid neural synchronization or mediator variable assessment, including measures such as DTF, PLV, coherence, oscillatory amplitude, or brain-to-brain coupling, with psychometric or methodological justification.
6.	Statistically appropriate and transparent data analysis, including proper use of connectivity analysis, neural-signal preprocessing, time-frequency analysis, or behavioural performance statistics suitable for BCI research.
7.	Adjustment for relevant confounders, such as prior gaming expertise, fatigue, cognitive load, age, sex, handedness, learning effects, or baseline neural variability.

## 3 Results

**Figure 1:**
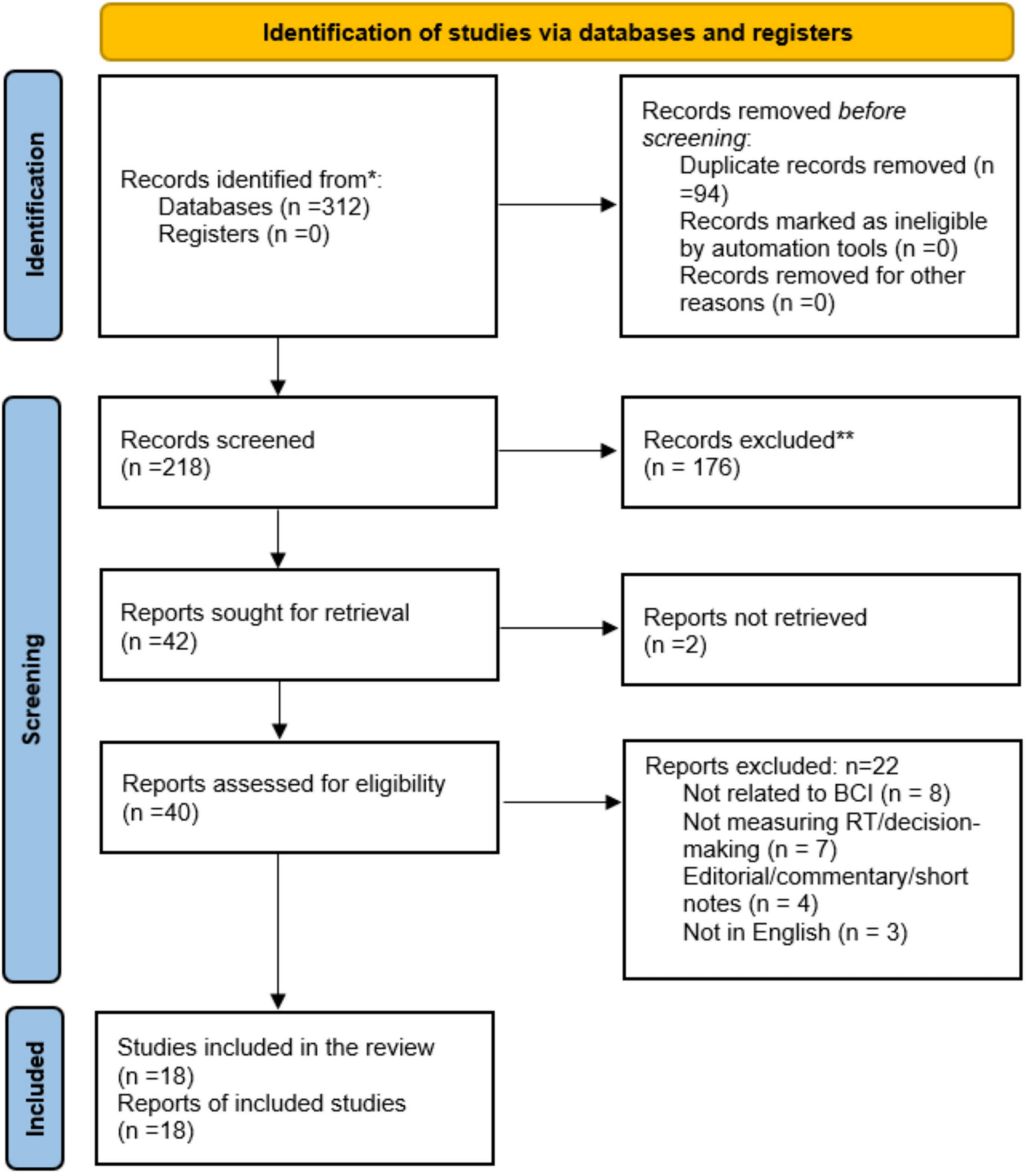
PRISMA 2020 flow diagram illustrating the study selection process. **Indicates studies excluded during full-text screening because they did not meet the eligibility criteria. *total records identified from databases/registers before duplicate removal and screening.

A total of 18 studies met the eligibility criteria and were included in the final synthesis. The body of evidence comprised experimental BCI interventions, cross-sectional neurophysiological investigations, comparative behavioural studies, and systematic reviews, reflecting the multidisciplinary nature of neural synchronization and esports performance research. Collectively, the studies evaluated a broad spectrum of outcomes, including reaction time, decision-making accuracy, neural connectivity and oscillatory dynamics, motor imagery-based BCI learning, cognitive functions such as working memory and inhibition, and motor-cognitive skills such as eye-hand coordination and visual fixation stability. Across the included studies, methodological quality varied, with controlled BCI experiments generally demonstrating lower risk of bias and observational studies showing moderate to serious concerns. Overall, the synthesis indicates that neural synchronization training, MI-BCI learning, and VEP/c-VEP-c-VEP-based systems consistently contribute to improvements in cognitive and performance-related parameters in esports and gaming populations, with multiple studies reporting faster reaction times, enhanced decision-making processes, stronger neural coupling, and greater cognitive efficiency (Figure 1 and Tables 3–10).

**Table 3:** Study characteristics.

Author (year)	Country/Region	Study design	Sample/Participants	BCI/Neural method	Intervention/Focus	Outcome measures	Key findings/relevance
Atilla et al. (2024)	Netherlands	Systematic Review	86 empirical studies published between 2012 and 2022 were identified and analyzed	MI-BCI, with all included studies utilizing Electroencephalography (EEG) for signal acquisition	Gamification of MI-BCI protocols	User performance, user experience, imagery ability	Gamified, user-centred BCI training using multimodal feedback, first-person humanoid avatars, adaptive difficulty, and social interaction enhances user performance, motivation, and experience while reducing training monotony.
Colzato et al. (2017)	Europe (Netherlands & Germany collaboration)	Narrative Review	Multiple experimental studies involving athletes and non-athletes	Noninvasive brain stimulation (tDCS), transcranial alternating current stimulation, and neurofeedback	Examination of neural stimulation and entrainment techniques to enhance athletic performance	Motor learning, muscle endurance, fatigue resistance, motion perception, sport-specific performance	Evidence suggests noninvasive brain stimulation and neural entrainment may enhance athletic performance by improving motor learning, muscle endurance, motion perception, and reducing fatigue, though further research with larger samples is required.
Gao et al. (2022)	China	Systematic Review	28 MI-BCI gaming studies involving 111 participants with accuracy testing	Non-invasive EEG-based Motor Imagery BCI	Gamification in MI-BCI training systems to improve user engagement and learning	Classification accuracy, game performance scores, and user experience metrics	Gamified MI-BCI training demonstrated an average accuracy of 74.35%, with 26 of 28 studies reporting positive outcomes, indicating that gamification can enhance engagement, reduce training monotony, and improve MI-BCI learning performance.
Jia et al. (2024)	China	experimental hyperscanning design. A multilevel modeling approach was employed to analyze interbrain functional connectivity	The study recruited 12 pairs of friends for the experimental group (24 healthy participants) and 10 pairs of strangers for the control group (20 healthy participants)	BCI/Neural Method: MI-based EEG BCI using 64-channel EEG hyperscanning, with Common Spatial Pattern (CSP) for feature extraction and Linear Discriminant Analysis (LDA) for classification.	The research focused on how social interaction factors, specifically eye contact and hand-touch, influence brain-to-brain coupling and neural synchronization during BCI training tasks	event-related desynchronization (ERD) to measure cortical activation, interbrain functional connectivity (using the Circular Correlation Coefficient), and BCI decoding accuracy	Social interaction between familiar users significantly improved MI-BCI decoding accuracy (≈78–79% vs. 70.7%), enhanced ERD and interbrain coupling, suggesting that familiarity-driven neural synchronization can improve BCI performance and may benefit robot-assisted rehabilitation.
Keutayeva et al. (2025)	Kazakhstan	Systematic Review	46 studies selected from 16,347 records	EEG-based VEP BCI including SSVEP, c-VEP, m-VEP, and t-VEP paradigms	Application of VEP-based BCIs in gaming environments to enable brain-controlled interaction and accessibility	Information Transfer Rate (ITR), classification accuracy, signal-to-noise ratio, user fatigue, and gameplay performance	VEP-based EEG BCIs enable high-speed, hands-free game control and improved accessibility, though challenges remain related to signal processing complexity, user comfort, and visual fatigue.
Li and Smith (2021)	USA	Systematic Review	28 studies (*N =* 829 participants; experimental group *n =* 430)	Functional neuroimaging techniques including EEG, fMRI, and fNIRS	Examination of the neural efficiency hypothesis in athletes during sport-specific perceptual–cognitive tasks	Cortical activation patterns (e.g., ERD/ERS, BOLD signals), reaction time, and task performance	Findings indicate that expert athletes generally show greater performance with reduced cortical activation**, s**uggesting more efficient neural processing due to long-term sport-specific training synchronisation
Minami et al. (2023)	Japan	Experimental study using a fighting video game (Street Fighter V) with a two-round first-pass system, combining physiological measurements and questionnaires	20 expert male players (mean age 24.4) ranked as “Grand Master” or higher	Mobile EEG (63 electrodes) focusing on frontal areas; analysis included cluster-based permutation tests and time-frequency multitaper spectral	Investigating how frontal cortex neural activity during pre-round periods contributes to the outcome of complex, prolonged e-sport matches	Match outcome (Win/Loss), subjective success levels of strategy/emotion, neural oscillation power (gamma, alpha, beta), heart rate (ECG), and arm muscle activity (EMG)	Increased frontal gamma (strategic decisions) and alpha power (emotional control) were associated with optimal performance, indicating frontal neural activity as a biomarker of competitive success.
Palaus et al. (2017)	Spain & USA	Systematic Review	116 studies involving >3,880 participants	Neuroimaging methods including EEG, fMRI, MRI, PET, NIRS, and SSVEP	Examination of neural correlates of video gaming and its cognitive effects	Brain activation patterns, structural brain changes, attention, cognitive control, visuospatial skills, reward processing	Video gaming is associated with functional and structural brain changes in prefrontal, parietal, and limbic regions linked to attention, cognitive control, visuospatial processing, and reward mechanisms.
Putri et al. (2022)	UK	directional connectivity analysis	10 dyads (20 participants total) with a mean age of 26.9 ± 4.7 years (8 females, 12 males)	EEG using 16 electrode locations. The control signal was the Relative Alpha (RA) band power (8–13 Hz) recorded at electrode location Pz. Connectivity was analyzed via Granger causality (GC) and Directed Transfer Function (DTF)	A multiuser competitive game based on neurofeedback where players controlled a virtual seesaw. The goal was to maintain a higher RA than the opponent to “lower” the seesaw and score points	RA power (Pz), Individual Alpha peak frequency, gaming scores, intra- and interbrain connectivity, and perceived workload (NASA-TLX).	Higher baseline alpha predicted better performance; winners showed effective Pz-driven brain connectivity and better opponent monitoring, while losers displayed inefficient frontal strategies without increasing alpha above baseline.
Yakovlev et al. (2020)	Russia	An experimental study using two subgroups (experimental and control) with pretest and posttest comparisons	14 healthy naive volunteers; the experimental subgroup included 8 participants, while the control subgroup included 6	A non-invasive EEG-based BCI was used to monitor mu-rhythm (7–13 Hz) event-related desynchronization (ERD). The system utilized a 30-channel EEG to provide visual neurofeedback based on sensorimotor activation	BCI-controlled kinesthetic MI training. The intervention focused on improving e-sports performance, specifically reaction time and velocity in mouse control	The primary measures were reaction time (measured in a speed selection task) and reaction velocity (measured in a one-minute fast-clicking task). EEG data were also analyzed to track ERD patterns during training	MI-based BCI training significantly improved reaction time and velocity with stable mu-ERD in all experimental participants, indicating enhanced fine motor control and fatigue resistance in professional e-athletes.
Cyma-Wejchenig et al. (2024)	Poland	Cross-sectional study	61 healthy young adults (aged 19–30), consisting of 36 non-professional esports players (EPs) and 25 non-players (NPs)	The study did not utilize a BCI. Instead, it assessed motor skills and cognitive processing speeds via a computer-based reaction time test (Mouse-Accuracy.com) and evaluated physical activity using the Baecke Questionnaire	Comparison of physical activity, esports reaction time, self-rated health, and injury prevalence between non-professional esports players and non-players to examine cognitive benefits versus sedentary health risks.	Physical activity indices (work, sport, leisure, total), reaction time metrics (clicks, hits, misses, accuracy, score), and self-reported health, gaming habits, screen time, seating type, and injury/pain prevalence.	Esports players showed faster reaction times but significantly lower physical activity and more gameplay-related pain, highlighting cognitive advantages alongside sedentary lifestyle risks and the need for ergonomic and exercise-based health strategies.
Ziv et al. (2022)	Israel & Belgium	Pre-registered, double-blinded, randomized online behavioral experiment	131 participants (out of an initial 187) who met criteria as gamers (*n =* 73, playing >10 h/week) or non-gamers (*n =* 58, playing <3 h/week). The final sample included 103 males and 27 females with a mean age of 23.51 years	The study utilized computerized behavioral tasks performed on the participants’ own computers; no Brain-Computer Interface or direct neural recording methods were employed	The focus was the timing of a video-game habits questionnaire (delivered either before or after cognitive tasks) to investigate if it created a response expectancy effect or psychological suggestion based on the participant’s status as a gamer or non-gamer	Mean Reaction Time and mean correct responses for a Choice-RT task, a Simon task, and an alternate task-switching task; and performance on a digit span memory task (total correct and highest digits remembered)	Questionnaire priming improved gamers’ reaction times but slowed non-gamers, indicating that psychological expectancy effects can bias gaming performance research outcomes.
Tmienova and Mykhalchuk (2023)	Ukraine	Experimental BCI training study	The study involves students and employees (referred to as decision-makers) in training and professional development scenarios. Specific session data presented in the source compares a participant’s results against a **20–**29 age group	BCI/Neural Method: Non-invasive EEG using the EMOTIV Insight 2.0 headset (AF3, AF4, T7, T8, Pz) with ERP detection and analysis via ICA, SVM, and CNN using EmotivPRO, BrainViz, and EMOTIV Labs.	The research focuses on reducing cognitive biases—categorized into perception, attention, memory, and motivation—to optimize decision-making and control. The primary training task used to investigate these biases is based on the Stroop effect	Evaluation indices include performance metrics (attention, engagement, and cognitive stress), behavioral performance (accuracy and reaction time), and emotional metrics (excitement, focus, and relaxation)	BCI-based neuroplasticity training enabled real-time evaluation of cognitive biases, with strongest correlations among attention, perception, and motivation, indicating its potential to enhance cognitive skills and professional efficiency.
Moreno-Calderón et al. (2023)	Spain	Experimental BCI Study	22 healthy controls (10 females, 12 males) with a mean age of 28 ± 2.60 years. Six participants had prior experience with BCI systems	c-VEP–based 8-channel EEG BCI using a 63-bit m-sequence circular shifting paradigm (120 Hz) with Canonical Correlation Analysis (CCA) for signal decoding.	Design and evaluation of a c-VEP-based BCI system in a competitive multiplayer gaming environment to examine its feasibility and the influence of competitiveness and motivation on BCI control.	Selection accuracy, information transfer rate (ITR), output characters per minute (OCM), selection time, workload (NASA-TLX), and usability (SUS).	The c-VEP BCI achieved high performance (93.7% accuracy; ITR ≈ 30.6 bpm) with low workload and high usability, outperforming SMR and P300 systems, demonstrating its effectiveness for competitive multiplayer BCI gaming.
Mancı et al. (2024a)	Turkey	Comparative cross-sectional study	39 esports players (MOBA *n =* 17; FPS *n =* 22)	Not applicable (behavioral cognitive assessment)	Comparison of cognitive performance between MOBA and FPS esports players	Change Detection, Mackworth Clock, Timewall, and Flanker tests measuring attention, working memory, reaction time, and inhibitory control	FPS players showed significantly better sustained attention, vigilance, reaction time, and inhibitory control than MOBA players, indicating genre-specific cognitive advantages in esports.
Velichkovsky et al. (2019)	Russia	experimental comparative analysis	The study included 21 participants categorized into three groups: low skill (10 amateurs with < 700 h of experience), high skill (7 amateurs with > 700 h), and professional (4 athletes from the Monolith team with > 10,000 h of experience)	While not a direct Brain-Computer Interface, the study utilized physiological and behavioral sensing, specifically a Tobii EyeX eye-tracker (measuring gaze at 30 Hz) and a Garmin Heart Rate Monitor (HRM) belt. Fixations were extracted from raw data using the Mould algorithm in the R statistical environment	The research focused on analyzing visual fixation durations during a realistic, emotion-provoking gaming session of Counter-Strike: Global Offensive (CS: GO) using a “Retake” modification scenario	Descriptive statistics (mean, median, SD, min–max), Vincentile analysis across five bins, and Kernel Density Estimation to examine unimodal or bimodal distribution patterns.	Expert players showed bimodal fixation patterns with short ambient and longer focal fixations, including very long (>500 ms) fixations absent in novices, suggesting eye-tracking patterns can inform adaptive interfaces based on skill level and cognitive load.
Lachowicz et al. (2024)	Poland	randomized controlled trial	The study included 66 amateur e-athletes (45 men and 21 women) with a mean age of approximately 24 years	The study utilized Virtual Reality (VR) technology, specifically the Valve Index VR headset. While the researchers did not directly measure neural changes, they discussed neuroplasticity and structural changes in the brain’s motor cortex as the likely underlying mechanisms for the observed improvements	Participants in the experimental group completed 15-min daily VR training sessions using Beat Saber for 8 consecutive days, with difficulty progressing from normal to expert level to increase cognitive and physical challenge.	Eye–hand coordination (EHC) and total percent error duration (TPED) via the S1 2HAND test, reaction time and motor time (MT) via the S2 Reaction Test, and VR side effects via the Simulator Sickness Questionnaire (SSQ).	Short-term VR training significantly improved eye–hand coordination and accuracy with effects maintained at 31-day follow-up, suggesting VR gaming can enhance esports-relevant cognitive–motor skills despite no changes in reaction or motor time.
Mancı et al. (2024b)	Turkey	A between-subjects design with pretest-posttest comparisons	19 healthy male participants aged 20–35, consisting of 10 amateur esports players and 9 age-matched controls who did not play esports	Functional near-infrared spectroscopy (fNIRS) was used to record cortical hemodynamic changes (oxy-hemoglobin, deoxy-hemoglobin, and total hemoglobin) in the prefrontal cortex (PFC)	The study focused on the effects of an acute bout of sprint exercise (SE) specifically a 30-s Wingate Anaerobic Test (WAnT) on the cognitive and gaming performance of participants	FPS gaming performance (time to destroy 50 targets), cognitive performance via Go/No-go and tracking tests, and prefrontal cortex hemodynamic activity during gameplay.	Esports players showed better baseline gaming and tracking performance; acute sprint exercise improved gameplay and inhibitory control accuracy without significant changes in PFC hemodynamics.

**Table 4:** Risk of bias assessment.

Study (author, year)	Study design type	Risk of bias tool used	Bias in selection/randomization	Bias due to deviations from intended intervention	Bias due to missing data	Bias in outcome measurement	Bias in selection of reported results	Overall Risk of bias judgment
Atilla et al. (2024)	Systematic Review	Not Applicable						Not Applicable
Colzato et al. (2017)	Review	Not Applicable						Not Applicable
Gao et al. (2022)	Review	Not Applicable						Not Applicable
Jia et al. (2024)	Controlled Experimental	RoB 2	Low	Low	Low	Low	Low	Low Risk
Keutayeva et al. (2025)	Systematic Review	Not Applicable						Not Applicable
Li and Smith (2021)	Systematic Review	Not Applicable						Not Applicable
Minami et al. (2023)	Cross-sectional Observational	ROBINS-I	Moderate	Low	Low	Low	Low	Moderate Risk
Palaus et al. (2017)	Systematic Review	Not Applicable						Not Applicable
Putri et al. (2022)	Observational Experimental	ROBINS-I	Moderate	Low	Low	Low	Low	Moderate Risk
Yakovlev et al. (2020)	Pre-Post BCI Training	RoB 2	Some concerns	Low	Low	Some concerns	Low	Some Concerns
Cyma-Wejchenig et al. (2024)	Cross-sectional	ROBINS-I	Moderate	Low	Low	Low	Low	Moderate Risk
Ziv et al. (2022)	Comparative Observational	ROBINS-I	Low	Low	Low	Low	Low	Low Risk
Tmienova and Mykhalchuk (2023)	Cognitive BCI Optimization	ROBINS-I	Moderate	Low	Low	Moderate	Low	Moderate Risk
Moreno-Calderón et al. (2023)	BCI Experimental	RoB 2	Some concerns	Low	Low	Low	Low	Low Risk
Mancı et al. (2024a)	Experimental (Exercise × Esports Cognition)	RoB 2	Some concerns	Low	Low	Some concerns	Low	Some Concerns
Velichkovsky et al. (2019)	Observational (Eye-tracking)	ROBINS-I	Serious	Low	Low	Low	Low	Serious Risk
Lachowicz et al. (2024)	Pre-Post VR Cognitive Training	RoB 2	Low	Low	Low	Low	Low	Low Risk
Mancı et al. (2024b)	Comparative Observational	ROBINS-I	Moderate	Low	Low	Low	Low	Moderate Risk

This table outlines the risk of bias ratings for each study based on RoB 2 or ROBINS-I criteria, indicating overall methodological quality. Risk of bias assessment was applied only to primary empirical studies. Systematic reviews and narrative reviews were marked as “Not applicable” because they were included for contextual background rather than primary data analysis.

**Table 5:** Reaction time outcomes across included studies.

Study	Population	BCI/Method	RT measure used	Main findings
Yakovlev et al. (2020)	Gamers	MI-BCI	Simple RT task	Significant RT reduction post-training
Jia et al. (2024)	BCI users	Brain-to-brain coupling	BCI task RT	Improved RT with neural synchronization
Cyma-Wejchenig et al. (2024)	Amateur esports	Standard RT test	E-game specific RT	Faster RT in high-activity esports players
Ziv et al. (2022)	Gamers vs. non-gamers	Behavioral test	Simple/choice RT	Gamers have significantly faster RT
Lachowicz et al. (2024)	Amateur esports	VR cognitive training	RT & eye-hand RT	RT improved after VR intervention
Mancı et al. (2024a)	FPS vs. MOBA players	Behavioral task	Choice RT	FPS players are faster RT

**Table 6:** Decision-making accuracy outcomes.

Study	Population	BCI/Method	Decision-making measure	Main findings
Putri et al. (2022)	BCI gamers	EEG DTF Connectivity	Competitive decision events	Winners show superior decision-synchronisation
Tmienova and Mykhalchuk (2023)	Students	Cognitive BCI	Bias reduction task	BCI reduced decision biases
Jia et al. (2024)	BCI users	Brain-to-brain coupling	Cooperative decision accuracy	Higher neural coupling was associated with improved decision-making accuracy
Minami et al. (2023)	Esports athletes	EEG oscillations	Match decisions	Better decisions linked to oscillation strength

**Table 7:** Neural synchronisation measures.

Study	Neural synchronisation variable	BCI/EEG technique	Main findings
Putri et al. (2022)	Directional connectivity (DTF)	EEG 32-channel	Winners showed increased fronto-parietal coupling
Jia et al. (2024)	Brain-to-brain coupling	Dual BCI	Stronger neural coupling was associated with improved performance
Minami et al. (2023)	Alpha/Beta oscillations	EEG	Higher oscillatory amplitude was associated with better competition performance
Moreno-Calderón et al. (2023)	c-VEP synchronization	c-VEP BCI	Multiplayer synchrony improved accuracy
Keutayeva et al. (2025)	VEP latency & reliability	VEP-BCI	Improved VEP synchrony reduces RT

**Table 8:** MI-BCI learning and neural control.

Study	MI performance/learning variable	System	Main findings
Atilla et al. (2024)	MI learning rate	MI-BCI (gamified)	Gamification improves MI learning
Gao et al. (2022)	MI-BCI learning curve	MI-BCI	Gamification improves neural adaptation
Yakovlev et al. (2020)	MI control accuracy	EEG MI-BCI	Accuracy improved after training

**Table 9:** Cognitive outcomes (memory, attention, inhibition).

Study	Cognitive variable	Method	Main findings
Ziv et al. (2022)	Working memory	Memory tests	Gamers demonstrated superior working memory performance compared with non-gamers
Mancı et al. (2024b)	Attention/inhibition	Go/No-Go tasks	Players of first-person shooter (FPS) games showed greater attention and inhibitory control than multiplayer online battle arena (MOBA) players
Palaus et al. (2017)	Attention/EF	fMRI/EEG reviews	Video gaming improves attention
Atilla et al. (2024)	Cognitive load	MI-BCI cognitive metrics	Gamification decreases cognitive load

**Table 10:** Motor-cognitive variables (eye-hand + visual fixations).

Study	Variable	Method	Main findings
Velichkovsky et al. (2019)	Fixation duration	Eye-tracking	Longer and more stable visual fixations were associated with higher skill levels
Lachowicz et al. (2024)	Eye-hand coordination	VR-RT test	VR training improved coordination
Cyma-Wejchenig et al. (2024)	Health + RT	Coordination test	Higher PA → better coordination

Overall, the collective findings from the included studies indicate that neural synchronization training and BCI-based approaches demonstrate meaningful potential for enhancing core performance-related capacities in esports athletes and competitive gamers. Across diverse methodologies, improvements were consistently observed in reaction time, decision-making accuracy, neural connectivity patterns, motor imagery learning, attentional control, and motor-cognitive coordination. Although the magnitude and consistency of effects varied according to study design and methodological quality, the convergence of evidence points toward a positive association between neural modulation techniques and competitive gaming performance. These results highlight both the emerging value of BCI technologies within esports science and the need for more rigorous, controlled trials to fully establish causal pathways and optimize training protocols. Collectively, the evidence base provides a strong foundation for interpreting the theoretical and practical implications discussed in the following section.

## 4 Discussion

The purpose of this systematic review was to synthesize empirical evidence examining how neural synchronization training and BCI systems influence reaction time, decision-making, cognitive performance, and neurophysiological efficiency among esports athletes and competitive gamers. Collectively, the findings across the 18 included studies provide converging support for the role of BCI-based neural training approaches particularly MI BCIs, VEP systems, dual-brain coupling, and neural entrainment in enhancing performance-related outcomes in digital competitive environments. While the studies employed heterogeneous methods and varied considerably in design quality, the overall patterns demonstrate that targeted modulation of neural activity through BCI use yields measurable benefits for cognitive speed, accuracy, and neural efficiency (Colzato et al., 2017; Putri et al., 2022; Yakovlev et al., 2020; Jia et al., 2024; Keutayeva et al., 2025), all of which are central to competitive esports performance.

One of the most consistent findings across the reviewed literature relates to the enhancement of reaction time. Experimental BCI studies and comparative cognitive research converge in suggesting that neural synchronization training can contribute to faster information processing and response execution. For example, motor imagery BCI training has been shown to reduce reaction time and improve task accuracy through repeated activation of motor cortical networks. Similarly, studies examining esports players outside direct BCI interventions indicate that experienced gamers demonstrate faster reaction times compared with non-gamers. While these findings collectively suggest a meaningful association between neural training and processing speed, the magnitude of these effects varies across studies due to differences in training protocols, neural recording methods, and participant expertise levels. Multiple lines of evidence indicate that BCI training protocols lead to improvements in speeded cognitive responses. Yakovlev et al. (2020) provided direct experimental evidence demonstrating that MI-BCI training significantly reduced RT and improved task accuracy, suggesting that the repetitive activation and control of motor cortical networks may sharpen neural processing speed. Similarly, Jia et al. (2024) showed that dual-brain BCI synchronization not only facilitated communication between paired participants but also resulted in faster coordinated responses, underscoring the potential of inter-brain coupling to accelerate collective task performance. Complementing these findings, studies not involving traditional BCIs also highlighted the importance of neural adaptability in shaping RT outcomes. For instance, Cyma-Wejchenig et al. (2024) observed that amateur esports players engaging in higher levels of gaming activity exhibited faster e-sport-specific RTs, while Ziv et al. (2022) demonstrated that gamers outperform non-gamers in both simple and choice RT tasks. These converging findings support the idea that repeated cognitive demands inherent in gaming may prime neural circuits for faster information processing, which can be further optimized using BCI-based neural training. It is important to note, however, that “reaction time” is operationalized heterogeneously across the included studies. Measures range from simple reaction time tasks and choice reaction paradigms to BCI-specific response latencies, in-game performance metrics, and perceptual-motor response times. These constructs, while related, reflect different levels of cognitive processing, from basic sensorimotor speed to complex decision-linked response execution. Therefore, the apparent consistency in RT improvements should be interpreted cautiously, as it may reflect convergence across related but non-identical constructs rather than a single unified performance domain. It is important to note that reaction time outcomes across studies are operationalized differently, including simple reaction time, choice reaction time, BCI response latency, and perceptual-motor response measures. Although these outcomes show broadly consistent improvements, they reflect partially distinct cognitive and neural processes. Therefore, consistency across studies should be interpreted as convergence across related but not identical constructs. These differences reflect variation in underlying processes, ranging from basic sensorimotor speed to higher-order perceptual decision-making and cognitive control mechanisms.

Decision-making accuracy also emerged as a robust outcome influenced by neural synchronization interventions. Putri et al. (2022) showed that winners in BCI-based competitive gaming tasks exhibited stronger directional connectivity particularly fronto-parietal networks highlighting that neural synchrony plays a pivotal role in effective decision-making under pressure. Minami et al. (2023) found support for this neural-behavioural link by demonstrating that higher parietal alpha and beta oscillatory amplitude predicted superior competition performance, suggesting that stable oscillatory patterns facilitate efficient perceptual and executive processing. Evidence from cognitive optimization studies also supports the role of neural modulation in enhancing decision-making. Tmienova and Mykhalchuk (2023) showed that cognitive BCI tasks reduced decision biases and improved accuracy, suggesting that BCIs can strengthen prefrontal cognitive control processes central to inhibition, assessment, and choice under uncertainty. Jia et al. (2024) demonstrated that stronger brain-to-brain coupling was associated with higher cooperative decision accuracy, offering a novel perspective on how synchronized neural states facilitate joint problem-solving in digitally mediated environments. Similarly, the construct of “decision-making” across studies encompasses a broad spectrum of cognitive processes, including competitive BCI task decisions, bias-reduction paradigms, cooperative interaction accuracy, and inferred decision efficiency based on neural oscillatory patterns. These variations suggest that decision-making, as used in the current literature, represents a multidimensional construct rather than a single, unified cognitive outcome. Consequently, while evidence indicates improvement across studies, the degree of conceptual coherence underlying these findings remains limited. In the present review, decision-making is used as a broad umbrella term encompassing multiple constructs, including behavioral outcomes (e.g., competitive decision accuracy), cognitive processes (e.g., bias reduction and strategic choices), and neural correlates (e.g., connectivity and oscillatory dynamics). These dimensions may not represent identical cognitive mechanisms, and therefore findings should be interpreted in relation to the specific task contexts in which decision-making was assessed.

Beyond RT and DM, a notable body of evidence links BCI-based interventions to improvements in neural synchrony and cognitive efficiency. Many of the reviewed studies examined neural-level changes to better understand the mechanisms underlying behavioural improvements. For example, Putri et al. (2022) reported that enhanced directional connectivity in winners of BCI competitions reflected more efficient neural communication pathways. Minami et al. (2023) further emphasized that oscillatory amplitude variations, especially in the parietal cortex, are strongly associated with high-level esports performance, pointing to the possibility that stable oscillatory rhythms may provide the neurophysiological foundation for fast, accurate in-game decision-making. Jia et al. (2024) expanded these concepts by demonstrating that brain-to-brain coupling is associated with heightened synchronized task performance, suggesting that inter-brain synchronization may offer an additional dimension for multiplayer esports training.

Additional support for neural synchronization as a training mechanism comes from studies focusing on non-invasive brain stimulation and neurophysiological entrainment. Colzato et al. (2017) reviewed evidence on neural entrainment techniques such as tACS and other NIBS methods, concluding that externally induced synchronization can improve RT, decision accuracy, and overall cognitive control. Their findings reinforce the broader theoretical position that synchronizing intrinsic neural oscillations either through direct stimulation or BCI-mediated training can meaningfully enhance cognitive readiness and response efficacy. Likewise, in a broad review of VEP-based BCI systems, Keutayeva et al. (2025) found enhancements in VEP signal reliability and reductions in RT latency, demonstrating that visual-cortical synchronization specifically contributes to performance gains in visually demanding tasks such as esports.

Motor imagery-related BCI learning and neural control were also recurrent themes in the literature. Atilla et al. (2024) and Gao et al. (2022) both highlighted the role of gamification in improving MI-BCI learning rates, neural engagement, and cognitive load management. Gamification appears to enhance user motivation, attention, and adherence, resulting in greater accuracy and faster neural adaptation, important considerations for the design of BCI training environments for esports athletes. These findings align with Yakovlev et al. (2020) empirical evidence showing improved MI control accuracy following structured MI-BCI training sessions. Collectively, these studies suggest that MI-BCI systems, particularly when integrated with game-like features, may provide a valuable training tool for improving neural control skills relevant to complex motor and perceptual tasks in esports.

Cognitive performance outcomes, including working memory, sustained attention, inhibition, and executive functioning, were also influenced by BCI training and gaming experience. Ziv et al. (2022) demonstrated that gamers exhibit superior working memory compared to non-gamers, reinforcing the idea that repeated cognitive challenges inherent in esports cultivate higher-order processing abilities. Mancı et al. (2024b) found that FPS players showed superior attention and inhibitory control relative to MOBA players, suggesting that different game genres impose distinct cognitive demands that shape attentional and executive profiles. Palaus et al. (2017) in a comprehensive review, highlighted that video gaming broadly enhances attentional capacity, visuomotor coordination, and executive functioning, all of which align with the cognitive requirements of high-performance esports. Atilla et al. (2024) also demonstrated that incorporating gamification into MI-BCI protocols reduces cognitive load, allowing users to maintain engagement and perform tasks with greater cognitive efficiency. Taken together, these findings suggest that both gaming experience and BCI-based cognitive training can enhance the cognitive resources necessary for optimal esports performance.

Motor-cognitive integration variables, such as hand-eye coordination and visual fixation stability, emerged as additional components of neural efficiency in gaming contexts. Lachowicz et al. (2024) reported that VR-based cognitive-motor training improved both RT and hand-eye coordination, demonstrating that immersive digital environments can facilitate sensorimotor learning relevant to esports. Velichkovsky et al. (2019) showed that longer, more stable visual fixations were associated with higher expertise levels among esports players, suggesting that perceptual efficiency and visual processing speed are key components of elite performance. Furthermore, Cyma-Wejchenig et al. (2024) found that players with higher physical activity levels exhibited better RT and coordination, indicating that general sensorimotor fitness may complement neural synchronization training for esports.

This review also identified several thematic patterns that help contextualize the overall findings. First, the integration of BCI systems into esports training appears most effective when interventions foster active neural adaptation whether through MI, VEP-based modulation, direct entrainment, or synchronized multiplayer environments. Second, successful neural synchronization training seems to rely on high-quality EEG signal acquisition, accurate classification algorithms, and well-structured protocols that promote user engagement. Third, although many studies report promising outcomes, methodological variability remains a challenge. Several observational studies were rated as having moderate or serious risk of bias, mainly due to non-randomized sampling, lack of control groups, or insufficient adjustment for confounders such as prior gaming experience or cognitive baseline differences.

Despite these limitations, the consistency of results across diverse methods suggests that improvements in RT, decision-making, and neural efficiency are not isolated artefacts but reflect genuine neural modulation effects (Colzato et al., 2017; Putri et al., 2022; Yakovlev et al., 2020; Jia et al., 2024; Keutayeva et al., 2025). The convergence of evidence across controlled experiments, neurophysiological studies, and gaming performance research strengthens the case for BCI-based approaches as emerging tools in esports training. Importantly, systematic reviews such as those by Atilla et al. (2024), Keutayeva et al. (2025), Li and Smith (2021), and Palaus et al. (2017) further support the theoretical framework linking neural adaptability, efficiency, and cognitive performance in competitive digital contexts. It is also important to acknowledge that the evidence base includes studies derived from a range of contexts, including esports athletes, general gaming populations, and non-gaming BCI or cognitive training paradigms. While these studies provide valuable mechanistic insights, their direct applicability to competitive esports performance varies. Therefore, conclusions regarding esports performance should be interpreted with consideration of this inferential distance, distinguishing between directly observed effects in esports contexts and those extrapolated from related domains.

### 4.1 Convergence and divergence of evidence

A key consideration emerging from this review is the differential consistency of findings across outcome domains. Reaction time and basic decision-making measures appear relatively more consistent across studies, potentially because these outcomes rely on simpler and more sensitive cognitive processes that are more directly influenced by neural modulation. In contrast, broader cognitive outcomes such as executive function, long-term adaptation, and transfer to real-world esports performance demonstrate greater variability. This divergence may be explained by several factors. First, complex cognitive constructs are assessed using diverse measurement tools, reducing comparability across studies. Second, variations in intervention duration, participant expertise, and BCI modality likely influence the magnitude and persistence of effects. Third, neural synchronization measures themselves vary widely, from oscillatory amplitude to connectivity metrics, limiting mechanistic consistency. Therefore, while convergence is observed at a general level, particularly for speeded cognitive processes, divergence remains substantial in higher-order and long-term outcomes, highlighting the need for more standardized methodologies and longitudinal research designs.

The relatively consistent findings for reaction time may be explained by the use of simpler, highly sensitive measurement paradigms that directly capture sensorimotor processing speed. In contrast, variability in higher-order cognitive outcomes likely reflects the complexity of these constructs, which require longer training durations and are assessed using diverse methodologies. Additionally, neural findings show variability due to differences in synchronization metrics, recording techniques, and analytical approaches.

Moving forward, future studies should aim to incorporate more rigorous experimental designs, larger sample sizes, standardized BCI training protocols, and long-term follow-up assessments. There is also a need for greater exploration of individual differences in neural plasticity, motivation, and learning curves, as these factors may shape responsiveness to BCI interventions (Atilla et al., 2024; Keutayeva et al., 2025). Additionally, integrating multimodal neuroimaging techniques such as fNIRS used by Mancı et al. (2024a) with EEG-based BCIs could provide richer insights into cortical dynamics underlying performance improvements. Overall, the evidence suggests stronger consistency in basic performance measures such as reaction time, while greater variability is observed in complex cognitive and neural outcomes, reflecting differences in task complexity, measurement approaches, and study designs.

### 4.2 Unresolved questions and future directions

Despite promising findings, several key questions remain unresolved in the current literature. These include whether different BCI modalities operate through shared or distinct neural mechanisms, the extent to which neural changes observed during BCI tasks transfer to real-world esports performance, and whether training effects are sustained over time. Additionally, it remains unclear how individual differences, such as expertise level, cognitive baseline, or game genre, influence responsiveness to BCI-based interventions. Furthermore, the diversity of neural metrics used across studies raises questions about whether they reflect a common dimension of performance readiness or distinct neurocognitive processes. Addressing these issues will be essential for advancing both theoretical understanding and applied implementation.

The evidence reviewed highlights the promising potential of neural synchronization training and BCI technologies for enhancing reaction time, decision-making, neural efficiency, and cognitive performance in esports athletes. While methodological limitations remain, the overall body of research provides a strong foundation for the continued development of BCI-based performance enhancement strategies in competitive gaming. Overall, evidence linking BCI-based neural synchronization with improvements in reaction time and decision-making appears relatively robust across multiple study designs. In contrast, findings concerning broader cognitive enhancements, long-term neural adaptation, and transfer of training effects to real-world esports performance remain less conclusive and require further investigation through larger and more controlled studies. Notably, findings related to reaction time improvements are supported by several low-risk experimental studies, strengthening confidence in these outcomes. In contrast, evidence concerning broader cognitive transfer and long-term adaptation is often derived from studies with moderate risk of bias or small sample sizes, and should therefore be interpreted with greater caution.

Despite the generally positive findings reported across the literature, several inconsistencies remain that warrant careful interpretation. Differences in BCI modalities, neural measurement techniques, training duration, and participant expertise levels introduce substantial methodological variability across studies. In addition, many investigations rely on relatively small samples or short-term interventions, limiting the ability to establish robust causal relationships between neural synchronization training and esports performance outcomes. Consequently, while the evidence supporting improvements in reaction time and decision-making appears relatively consistent, conclusions regarding broader cognitive adaptations and long-term neural plasticity remain more tentative. The interpretation of findings must also be considered in light of study quality. Domains showing relatively consistent effects, such as reaction time, are supported by a mixture of low-risk experimental studies and moderate-risk observational evidence. In contrast, areas such as cognitive transfer and neural adaptation are often derived from studies with smaller samples or a higher risk of bias, contributing to the observed uncertainty. Thus, the strength of conclusions across domains reflects not only conceptual consistency but also variation in methodological quality. While several findings are supported by direct esports-related studies, others are derived from broader BCI and cognitive neuroscience research and should be interpreted with consideration of this contextual difference.

### 4.3 Limitations

This systematic review offers valuable insights, but several limitations must be noted. The included studies exhibited considerable methodological heterogeneity, with wide variations in BCI modalities, neural measures, and performance assessments, limiting opportunities for meta-analysis. Many studies used small samples or cross-sectional designs, reducing statistical power and preventing strong causal inference. Risk of bias was moderate to high in several observational studies due to non-random sampling, inadequate control of confounding variables, and limited reporting of EEG preprocessing. Technological differences across BCI systems and esports genres further contributed to inconsistent results. Additionally, most studies focused on short-term effects, leaving long-term training impact and real-world applicability largely unexplored.

### 4.4 Practical implications for coaches and trainers

The findings of this review offer several actionable insights for esports coaches and performance trainers. First, incorporating BCI-based neural synchronization training into routine practice sessions may have the potential to enhance reaction speed, decision-making precision, and attentional control skills that directly translate into in-game performance advantages. Motor imagery BCIs, VEP-based systems, and synchronized dual-brain tasks can be used as structured warm-up or cognitive activation tools to prepare players for high-intensity competitive environments. Second, neural markers such as oscillatory amplitude, connectivity patterns, and fixation stability may serve as objective indicators of player readiness, fatigue, or cognitive overload, allowing coaches to individualize training loads and recovery strategies. Third, gamified BCI tools can support motivation, reduce cognitive fatigue, and encourage consistent practice, making them suitable for both amateur and professional training environments. Finally, integrating neurocognitive assessments into player development programs can help identify strengths and weaknesses not detectable through gameplay analysis alone, enabling more targeted and efficient skill development. However, these applications should be considered preliminary and contingent upon further validation through large-scale and longitudinal experimental studies.

### 4.5 Theoretical implications for neural synchronization research

This review contributes to the theoretical advancement of neural synchronization research by demonstrating its close relationship with performance outcomes in fast-paced cognitive environments such as esports. The consistent association between fronto-parietal connectivity, oscillatory stability, and decision accuracy provides empirical support for models positing that synchronized neural activity underpins efficient information transfer and executive control. Findings from dual-brain BCI studies also expand existing theories by showing that synchronized neural states can emerge not only within individuals but also between interacting players, suggesting that team-based neural coupling may influence cooperative performance. Furthermore, improvements in motor imagery control, visual evoked potentials, and oscillatory tuning emphasize the plasticity of neural networks involved in attention, perception, and motor planning. These results collectively support emerging frameworks that conceptualize BCIs as tools for adaptive neural modulation, where repeated training drives cortical reorganization, improved synchronized firing, and enhanced system-level efficiency.

### 4.6 Implications for future BCI system design

The results of this review hold significant implications for the development of next-generation BCI systems for performance enhancement. Future BCIs should prioritize high signal quality and stability by incorporating improved artifact rejection, adaptive filtering, and more robust classification algorithms capable of handling fast-paced, high-noise esports environments. Gamification emerges as a key design principle, as studies consistently showed that game-like interfaces enhance engagement, reduce cognitive load, and accelerate MI-BCI learning. BCIs may also benefit from integrating multimodal sensing combining EEG with fNIRS, eye-tracking, or physiological measures to capture a more comprehensive picture of cognitive states. The success of dual-brain coupling systems suggests future potential for team-based BCIs designed to train coordination, communication, and shared situational awareness in multiplayer esports. Additionally, systems should be designed for short, repeatable training sessions that fit seamlessly into esports practice schedules, with adaptive difficulty levels to account for individual variability in learning curves and neural plasticity. Ultimately, the convergence of usability, engagement, and neuroscientific precision will be essential in creating BCIs that are both performance-enhancing and practical for real-world esports training.

## 5 Conclusion

Overall, the available evidence suggests a generally positive association between BCI-mediated neural synchronization and improvements in reaction time, decision-making, and selected cognitive outcomes. However, this evidence remains heterogeneous in methodology and scope, with varying levels of study quality and limited long-term validation. Therefore, while the findings are promising, they should be interpreted with appropriate caution, particularly regarding broader cognitive generalization and real-world esports performance transfer. Although heterogeneity in study designs, small sample sizes, and varying methodological quality limited the strength of causal inference, the converging findings highlight BCIs as promising tools for both cognitive enhancement and performance optimization in esports. These results underscore the value of integrating neuroscience-driven training methods into competitive gaming environments and point to the growing relevance of neural synchronization as a theoretical mechanism for understanding expert performance. Future research should prioritize larger, well-controlled trials, standardized BCI training protocols, and long-term monitoring to fully understand the durability, transferability, and practical applicability of neural modulation techniques in real-world esports settings. Some conclusions are based on adjacent evidence from general gaming and BCI research, and therefore direct evidence specific to esports remains comparatively limited. Overall, this review establishes a strong foundation for continued exploration of BCIs as innovative, evidence-based interventions capable of advancing both the science and practice of esports performance enhancement.

## Data Availability

The data analyzed in this study is subject to the following licenses/restrictions: all evidence presented in this systematic review is sourced from previously published research. No original datasets were created or analysed for this study. Complete details of the included studies, including extraction tables and methodological documentation, can be obtained from the corresponding author upon reasonable request. Requests to access these datasets should be directed to PC, prashantlnipe2014@gmail.com.
